# Hyaluronidase recruits mesenchymal-like cells to the lung and ameliorates fibrosis

**DOI:** 10.1186/1755-1536-4-3

**Published:** 2011-01-13

**Authors:** Claudia S Bitencourt, Priscilla AT Pereira, Simone G Ramos, Suely V Sampaio, Eliane C Arantes, David M Aronoff, Lúcia H Faccioli

**Affiliations:** 1 Departamento de Análises Clínicas, Toxicológicas e Bromatológicas, Faculdade de Ciências Farmacêuticas de Ribeirão Preto, Universidade de São Paulo, Ribeirão Preto, SP, 14040-903, Brazil; 2 Departamento de Patologia, Faculdade de Medicina de Ribeirão Preto, Universidade de São Paulo, Ribeirão Preto, SP, 14049-900, Brazil; 3 Departamento de Física e Química, Faculdade de Ciências Farmacêuticas de Ribeirão Preto, Universidade de São Paulo, Ribeirão Preto, SP, 14040-903, Brazil; 4 Division of Infectious Diseases, Department of Internal Medicine, and the Department of Microbiology and Immunology, University of Michigan Health System, Ann Arbor, MI 48109, USA

## Abstract

Hyaluronidases (HYALs) comprise a group of enzymes that degrade hyaluronic acid (HA). In this report, we reveal that a single intranasal inoculation of HYAL induces an increase in mononuclear cells within the bronchoalveolar space demonstrating a mesenchymal-like phenotype, expressing stem cell antigen-1 (SCA-1), CD44 and CD73 but not CD34, CD45, CD3, CD4, CD8 or CD19. This influx of mesenchymal stem cell (MSC)-like cells was dependent on leukotriene production within the lung parenchyma. These findings prompted experiments demonstrating that HYAL treatment potently blocked bleomycin-induced lung injury and fibrosis while decreasing transforming growth factor (TGF)-β production and collagen deposition. These data suggest that HYAL is a novel and promising tool to use autologous MSC-like cells in the treatment of pulmonary fibrosis.

## Background

The hyaluronidases (HYALs) are a group of enzymes that regulate hyaluronic acid (HA) metabolism and consequently remodel the extracellular matrix (ECM) [1]. These enzymes are produced by: mammals as a component of seminal fluid, plasma and urine [1]; bacteria as a virulence factor [2,3]; and venomous animals as a non-toxic component of venoms [1]. HYALs have been used therapeutically due to their capacity to reduce biological fluid viscosity, increase vascular permeability and render tissues more accessible to certain drugs [4].

There is much interest in the HYAL-HA axis in the treatment of inflammatory disorders [1]. While many studies demonstrated the involvement of HA in inflammatory responses, the involvement of HYALs has been less well studied [5,6]. Although prior investigations have measured tissue HA levels or HYAL messenger RNA (mRNA) levels, few have directly assessed the effect of HYALs on cell or organ function *per se*.

Mesenchymal stem cells (MSCs) are pluripotent cells that can differentiate into a variety of cells, including osteoblasts, myocytes, adipocytes and chondrocytes. Recently, it has been demonstrated that MSC can differentiate along a non-stromal lineage to become lung epithelial cells [7]. The bone marrow is the principal source for MSCs [8,9] but these cells have also been isolated from the umbilical cord [10], fetal membranes [11] and other tissues. The use of MSCs to regenerate tissues has been reported as a promising therapy for the treatment of a variety of diseases [12,13]. However, cellular therapy using MSCs has obstacles, including: the difficulty of obtaining adequate numbers of these cells to transplant; adverse effects of the cells or concomitant immunosuppressive therapies; and the possibility of infection by opportunistic microorganisms [13-15].

Increasingly, MSC-based therapies have been considered a promising new means of treating chronic lung diseases such as pulmonary fibrosis, a progressive, highly-lethal disorder for which very few effective therapies exist [7,13]. For example, MSCs were shown to ameliorate bleomycin-induced lung fibrosis in mice, an effect that was associated with diminished lung damage and ECM collagen deposition within the lung [16]. In addition, MSCs obtained from the umbilical cord were recently reported to diminish bleomycin-induced fibrosis [10].

In the light of this, we sought to examine the actions of HYAL therapy on the lung microenvironment and on pulmonary inflammation. We investigated the effects of intranasal inoculation (i.n.) of HYAL isolated from either bovine testes or *Tityus serrulatus*, a yellow scorpion (HYAL-TS). These studies, for the first time, demonstrate that a single dose of HYAL increased a heterogeneous population of mononuclear cells with an MSC-like phenotype in bronchoalveolar fluid (BALF). In addition, HYAL therapy reduced bleomycin-induced fibrosis. These novel results suggest that HYAL therapy should be further examined as a strategy to treat pulmonary fibrosis.

## Results

### HYAL induces late and preferential increases in mononuclear cell numbers in the bronchoalveolar space

**Figure 1:**
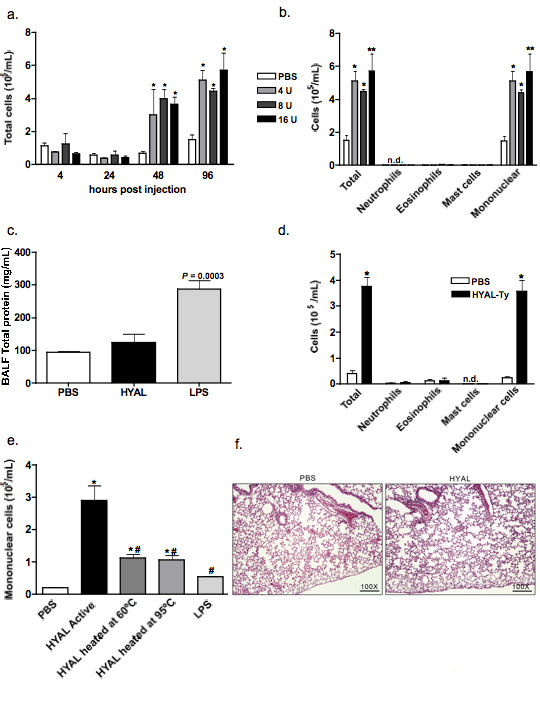
**Hyaluronidase (HYAL) induces an increase in leukocytes in bronchoalveolar fluid (BALF) of C57Bl/6 mice**. (A) Total cells obtained from bronchoalveolar space after 4, 24, 48 and 96 h of inoculation with 4, 8 or 16 U of bovine testicular HYAL. Values represent means ± standard error of mean (SEM); *n *= 4; * *P *< 0.05 compared to phosphate buffered saline (PBS); ANOVA test was used. (B) HYAL induces almost exclusively mononuclear cell increases in the bronchoalveolar space after 96 h of inoculation with 16 U of bovine testicular HYAL in C57Bl/6 mice. N.d = not detected. Values represent means ± SEM; *n *= 4; * *P *< 0.05, ** *P *< 0.01 compared to PBS by ANOVA. (C) 16 U HYAL did not induce oedema after 96 h. The total protein content was measured in BALF. Llopopolysaccharide was inoculated intranasally (i.n.) as a positive control of oedema (500 μg/μL in 20 μL). Values represent means ± SEM; *n *= 4; *P *= 0.003 *t *test compared to the PBS. (D) *Tityus serrulatus *hyaluronidase (HYAL-TS) also induced mononuclear increase in the bronchoalveolar space after i.n. inoculation of 16 U in C57Bl/6 mice. N.d = not detected. Values represent means ± SEM; *n *= 5; * *P *= 0.003 using test *t*, compared to the PBS. (E) HYAL inactivation by heating reduces mononuclear cell numbers in bronchoalveolar space. 16 U of bovine testicular HYAL were heated or not at 60°C or 95°C i.n. inoculated and BALF cells collected 96 h later. Values represent means ± SEM; *n *= 5; * *P *< 0.001 compared to PBS; # *P *< 0.001 compared to active HYAL by ANOVA. (F) Photomicrographs of representative lung sections obtained from mice inoculated with PBS or HYAL. Tissues were stained with H&E to investigate inflammatory cells accumulation. Original magnification: 100x.

In order to determine the effects of HYAL on the lung microenvironment, mice were inoculated intranasally with a single dose containing 4, 8 or 16 U of bovine testicular HYAL and cells present in BALF were recovered at different time points. Unexpectedly, we found that i.n. HYAL induced a late, and almost exclusive, mononuclear cell increase in BALF between 48 and 96 h after inoculation (Figure 1A and 1B). The maximal effect was observed at 4 U, with no further increase at 8 or 16 U. We then administered 16 U of HYAL and observed animals 96 h post inoculation. Interestingly, 96 h after the i.n. inoculation of HYAL, oedema formation was not observed, indicating that this enzyme did not induce an increase in lung vascular permeability at this time, in contrast to i.n. lipopolysaccharide (LPS) administration (Figure 1C). In order to determine whether the cellular increases in the lung were due to a specific effect of bovine testicular HYAL, we performed similar experiments with HYAL-TS. As we observed the same profile obtained using either HYAL or HYAL-TS, we used only testicular HYAL for future experiments (Figure 1D). In order to assess whether the increases in cell numbers in BALF were dependent on enzymatic activity *per se*, HYAL was heated and denatured at 60° or 95°C for 60 min, respectively, before inoculation. We observed a decrease of ~70% in mononuclear cells present in BALF, compared to the active enzyme (Figure 1E). However, heated and inactive HYAL still induced a significant increase in mononuclear cells in BALF compared to phosphate buffered saline (PBS). HYAL did not induce alterations in the lung architecture (Figure 1F).

### HYAL induces cell recruitment through a mechanism dependent on leukotrienes, prostaglandins and cytokines

**Figure 2:**
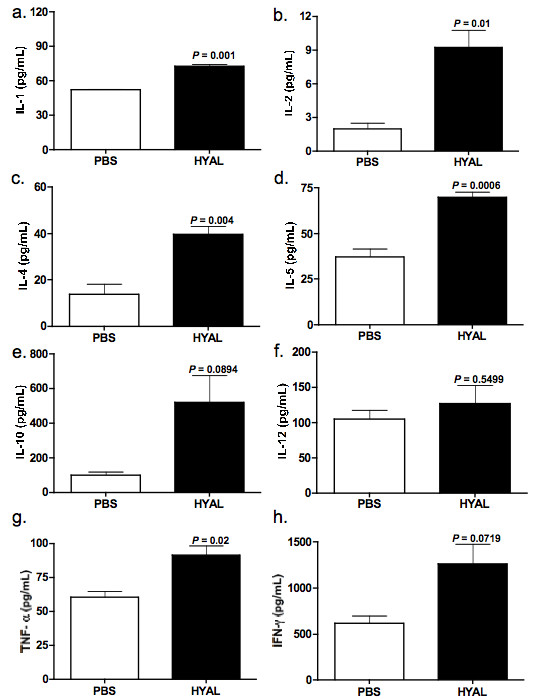
**Hyaluronidase (HYAL) modulates cytokines in the lung parenchyma**. Supernatants were prepared from the lung parenchyma obtained from C57Bl/6 mice treated after 96 h of inoculation with 16 U of bovine testicular HYAL or phosphate buffered saline (PBS). Interleukin (IL)-1, IL-2, IL-4, IL-5, IL-10, IL-12, tumour necrosis factor (TNF)-α and interferon (IFN)-γ were assayed by ELISA. (A) IL-1, *P *= 0.001; (B) IL-2, *P *= 0.01; (C) IL-4, *P *= 0.004; (D) IL-5, *P *= 0.006; (E) IL-10, *P *= 0.08; (F) IL-12, *P *= 0.54; (G) TNF-α, *P *= 0.02; (H) IFN-γ, *P *= 0.07; (A-H) = values represent means ± SEM compared to PBS; *n *= 5, *t *test was used.

**Figure 3:**
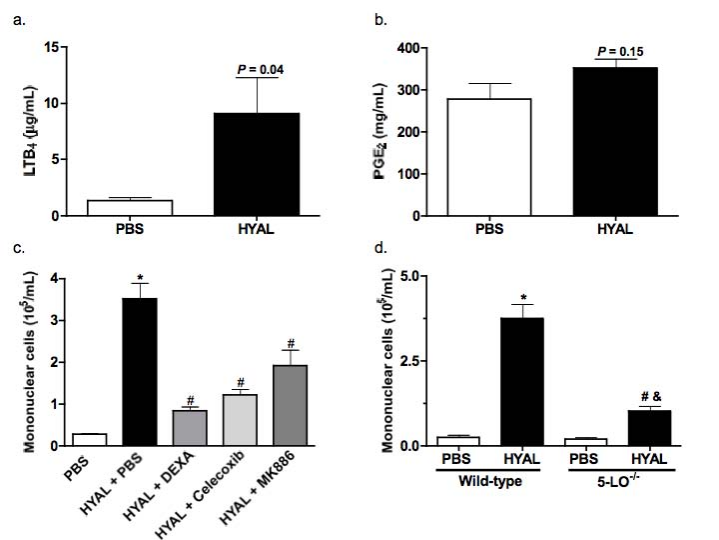
**Hyaluronidase (HYAL) induces mononuclear cell increase in bronchoalveolar space by mechanism partially dependent on lipid mediators**. (A) Leukotriene (LT)B_4 _presents in the supernatant of lung homogenates were analysed by specific electroimmunoassay. C57Bl/6 was inoculated intranasally (i.n.) with 16 U of bovine testicular HYAL or phosphate buffered saline (PBS) and the lungs were removed 96 h later. Values represent means ± standard error of mean (SEM); *n *= 10; *P *= 0.04 compared to the PBS; *t *test was used. (B) Prostaglandin E_2 _concentration in the supernatant of lung homogenates was analysed by specific electroimmunoassay. C57Bl/6 was inoculated i.n. with 16 U of bovine testicular HYAL or PBS and the lung was removed 96 h later. Values represent means ± SEM; *n *= 10; *P *= 0.15 compared to the PBS group, *t *test was used. (C) Pre-treatment with dexamethasone (1 mg/kg s.c.); MK886 (5 mg/kg/0.5 mL by gavage) or celecoxib (5 mg/kg/0.5 mL by gavage) significantly reduced the number of cells in bronchoalveolar space of C57Bl/6 mice. All treatments were performed 1 h before stimuli and every 24 h until the end of experiment. Control received only PBS. Values represent means ± SEM.; *n *= 5; * *P *> 0.01 compared to PBS; # *P *> 0.001 compared to HYAL + PBS, ANOVA test was used. (C) Mononuclear cells recruitment to the bronchoalveolar space of 5-LO ^-/- ^(129-Alox^5tm1Fun^) mice is decrease when compared to strain-matched wild-type (WT) mice. Each strain was inoculated with 16 U of HYAL and cells recovered 96 h after stimulus. Values represent means ± SEM; *n *= 5; * *P *< 0.0001 compared to PBS WT; ^# ^*P *= 0.0003 compared to PBS 5-LO ^-/-^; ^# ^*P *= 0.0003 compared to HYAL WT; test *t *was used.

Figure 2 illustrates that interleukin (IL)-1, IL-2, IL-4, IL-5, IL-10 and TNF-α are significantly increased 96 h after treatment with 16 U HYAL, suggesting that these cytokines might be involved in cell recruitment to the BALF (Figure 2A - H). As lung cells are a rich source of immunoregulatory lipid mediators, we also quantified the leukocyte chemoattracting eicosanoid leukotriene B_4 _(LTB_4_) in lung parenchyma. LTB_4 _was significantly increased compared to PBS (Figure 3A). Figure 3C shows mononuclear cell numbers recovered from BALF of mice treated with the leukotriene synthesis inhibitor MK886 or the anti-inflammatory compound dexamethasone. Treatment of mice with dexamethasone decreased the number of cells recovered from BALF by 76%, compared to those receiving HYAL alone. When compared to vehicle-treated animals, mononuclear cell numbers were significantly diminished by MK886 pre-treatment of HYAL-exposed mice (46%) or celecoxib pre-treatment of HYAL-exposed mice (65%), indicating the participation of leukotrienes and prostaglandins (PGs) in HYAL-induced cell migration (Figure 3C). To further implicate the involvement of PGs, we measured PGE_2 _concentrations, which tended to increase in lung homogenates after HYAL, but this result was not significant (Figure 3B). In order to confirm the involvement of leukotrienes in the mononuclear recruitment induced by HYAL, 5-LO ^-/- ^(129-Alox5^tm1Fun^) and strain-matched wild type (WT) were injected i.n. with HYAL. The mononuclear cells obtained from 5-LO ^-/- ^BALF after 96 h were significantly diminished in comparison to WT (Figure 3D). Additionally, other cell types were not altered by HYAL in 5-LO ^-/- ^and WT. These results suggest that HYAL-induced mononuclear cell recruitment is at least partially dependent on leukotriene, prostaglandin and cytokine production.

### Immunophenotypic characterization of mononuclear cells present in BALF

**Figure 4:**
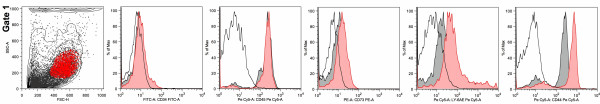
**Hyaluronidase (HYAL) induced an increase in bronchoalveolar cells with a phenotype of mesenchymal stem cells**. Gate 1 is a representative picture showing forward/side scatters dot-plot of bronchoalveolar fluid cells obtained from phosphate buffered saline (PBS) treatment (black contour-plot) overlaid upon HYAL exposure (grey dot-plot), as well as the gate population highlighted in red. Histograms overlays were done using the gated population highlighted in red; open histogram = isotype; grey filled histogram = PBS; red filled histogram = HYAL. The figure depicts a representative analysis from five independent experiments.

**Figure 5:**
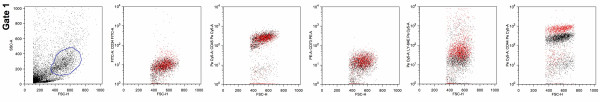
**Hyaluronidase (HYAL) induced an increase in bronchoalveolar cells with a phenotype of mesenchymal stem cells**. Gate 1 is a representative picture showing forward/side scatters dot-plot of bronchoalveolar cells obtained from PBS treatment (black) overlaid upon HYAL exposure (red). The figure depicts a representative analysis from five independent experiments.

**Figure 6:**
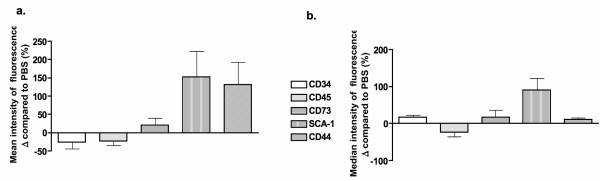
**Hyaluronidase (HYAL) induced an increase in bronchoalveolar cells with a phenotype of mesenchymal stem cells**. The mean (A) and median (B) expression of markers is altered by HYAL in gate 1. Phosphate buffered saline values were considered to be 100%. The figure depicts a representative analysis from five independent experiments.

**Figure 7:**
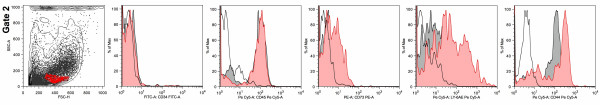
**Hyaluronidase (HYAL) induced an increase in bronchoalveolar cells with a phenotype of mesenchymal stem cells**. Gate 2 is a representative picture showing forward/side scatters dot-plot of bronchoalveolar fluid cells obtained from phosphate buffered saline (PBS) treatment (black contour-plot) overlaid upon HYAL exposure (grey dot-plot), as well as the gate highlighted in red. Histograms overlays were done using the gated population highlighted in red; open histogram = isotype; grey filled histogram = PBS; red filled histogram = HYAL. The figure depicts a representative analysis from five independent experiments.

**Figure 8:**
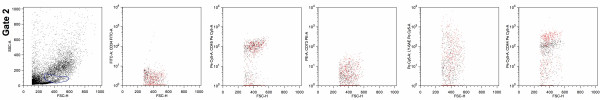
**Hyaluronidase (HYAL) induced an increase in bronchoalveolar cells with a phenotype of mesenchymal stem cells**. Gate 2 is a representative picture showing forward/side scatters dot-plot of bronchoalveolar fluid cells obtained from phosphate buffered saline treatment (black) overlaid upon HYAL exposure (red). Figure depicts a representative analysis from five independent experiments.

The populations of BALF cells obtained after HYAL inoculation were studied by flow cytometry and gated into four distinct sub-populations according to their forward/side scatter patterns. These sub-populations were referred to as gates 1, 2, 3 and 4. In two of these cell groups, we observed striking differences in cell numbers between vehicle (PBS) and HYAL-induced mononuclear cells (Figures 4, 5, 6, 7, 8). First, we performed analyses using anti-CD3, CD4, CD8, CD19, MAC-3 and CD11c immunoglobulins, in order to identify whether these cells were lymphocytes and/or macrophages. Surprisingly, the expression of these markers was not altered in bronchoalveolar cells after HYAL exposure. Some samples showed a decrease in fluorescence intensity, indicating that the cells that increased in the BALF were not CD4^+ ^T lymphocytes (CD3^+^/CD4^+^), CD8^+ ^T lymphocytes, B lymphocytes (CD19^+^) or macrophages (MAC3^+^/CD11c^-^) (see Additional File 1; Figure S1A - F). In addition, the decrease of these mature lineage markers suggested that the increased population of cells in the bronchoalveolar space might be immature or relatively undifferentiated.

In order to establish the nature of the cellular changes induced by HYAL exposure, flow cytometric analyses were performed using hematopoietic and non-hematopoietic markers on the four sub-populations described above. In gate 1 (gate 1 in Figures 4 and 5), following HYAL treatment, we observed a decreased in CD34 (42%) and CD45 (23%) expression, increased stem cell antigen-1 (SCA-1; 152%) and CD44 (132%) expression, followed by a marginal increase in CD73 (39%) compared to PBS-treated mice (Figure 6). The overlaid histograms showed significant changes in the expression of surface marker profiles comparing PBS and HYAL treatment, where CD44 and SCA-1 peaks were shifted to the right (Figure 4) as were the number of events (Figure 5).

**Figure 9:**
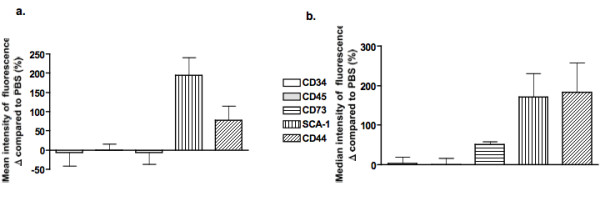
**Hyaluronidase (HYAL) induced an increase in bronchoalveolar cells with a phenotype of mesenchymal stem cells**. The mean (A) and median (B) expression of markers was altered by HYAL in gate 2, presented as the percentage of variation compared to the control. The controls mice were considered to be 100%. The figure depicts a representative analysis from five independent experiments.

The same expression profile was observed in gate 2 (Figures 7 and 8), but CD44 expression was decreased compared with gate 1. When compared to PBS, mean and median fluorescence intensities were similar (Figures 6 and 9), compatible with the related histograms. The other two gates (3 and 4) did not show the same profile of marker expression and were uninformative (see Additional File 2; Figure S2A - F).

Cells labelling positive for SCA-1, CD44, CD73 and negative for CD34 and CD45 have been described as murine MSCs. These data provide a basis from which we can postulate that the cells increased in BALF following HYAL treatment are also MSC-like cells.

### MSC-like cells are increased in lung ameliorate the bleomycin-induced fibrosis

**Figure 10:**
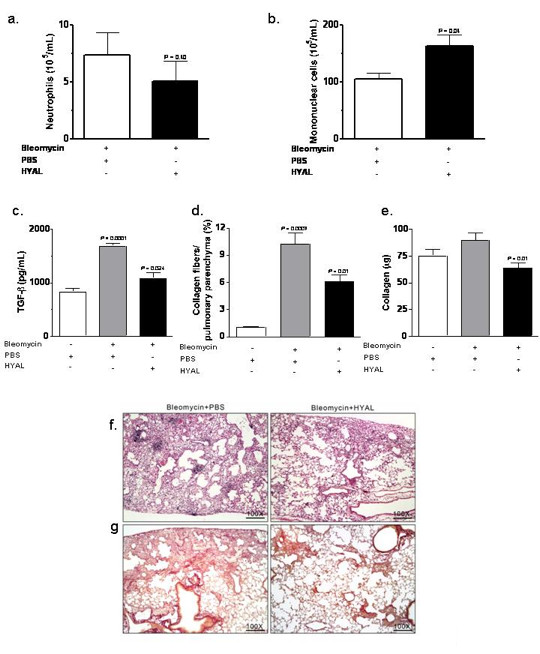
**Effects of hyaluronidase (HYAL) treatment on bleomycin-induced inflammation in the lung**. At day 8 after bleomycin treatment (4 U/kg/0.1 mL NaCl 0.9%) in the lungs of C57Bl/6 mice, animals were treated with intranasal inoculation (i.n.) once with 16 U HYAL or phosphate buffered saline (PBS) and inflammatory cells recovered 96 h after. (A) Neutrophils numbers in the bleomycin-inoculated lung were not altered by HYAL (*P *= 0.406). However, (B) mononuclear cells were significantly increased by HYAL treatment (*P *= 0.045). Values represent means ± standard error of mean (SEM).; *n *= 5, by *t *test (C) transforming growth factor (TGF)-β is increased by bleomycin but reduced by HYAL treatment. TGF-β was measured in supernatant of lung tissue homogenate by ELISA. Values represent means ± SEM; *n *= 5, *P *= 0.0024 compared to bleomycin, *P *< 0.0001 compared to PBS, by *t *test. (D) The picrosirius morphometric analysis corresponding to the area occupied by the fibres were determined by digital densitometry recognition and expressed as a percentage of the total area of the field. Values represent means ± SEM; *n *= 10; *P *= 0.010 compared to PBS treated, using *t *test. (E) Acid-soluble collagen in the supernatant of lung tissue homogenate, assayed by Sircol. Values represent means ± SEM; *n *= 10; *P *= 0.010 compared to PBS, by *t *test. (F, G) Photomicrographs of representative lung sections obtained from mice inoculated with bleomycin and treated with PBS or with HYAL. In (F) tissues were stained with haematoxylin and eosin in order to investigate inflammatory cells accumulation and in (G) with Picrosirius to determine the collagen content. The histological analyses were single-blinded. Original magnifications = 100x.

In light of the finding that the cells increased in the BALF of mice exposed to HYAL were phenotypically consistent with MSCs-like, we tested the potential of HYAL to mollify bleomycin-induced pulmonary fibrosis. Mice were exposed to bleomycin on day 0, and on the 7th day post-bleomycin, inflammatory cells were recovered from the bronchoalveolar space and enumerated in order to confirm the presence of inflammatory cells. As expected, bleomycin induced an inflammatory reaction with a high number of neutrophils and mononuclear cells compared to the PBS-inoculated control mice (see Additional File 3; Figure S3A and B). In addition, groups of mice inoculated with bleomycin were treated i.n. on the 8th day post-bleomycin with 16 U of HYAL or PBS vehicle and inflammatory responses were evaluated 96 h (4 days) later (Figure 10). This period of treatment corresponded to the same time that we observed an increase in MSC-like cells in the BALF of HYAL-inoculated mice. Interestingly, HYAL led to a non-significant reduction in neutrophil recruitment following bleomycin (Figure 10A), followed by a significant increase in mononuclear cell numbers (Figure 10B). Also, cytokines involved in the progression of fibrosis were evaluated. We did not observe alterations in MCP-1 or KC after HYAL treatment (see Additional File 3; Figure S3C and D). On the other hand, a significant reduction in TGF-β concentrations was observed in lung homogenates after HYAL treatment (Figure 10C).

Figures 10F and 10G illustrate the morphology of the lung after the inoculation of bleomycin and either HYAL or PBS treatment. Histopathological findings in the lungs of mice 12 days after of bleomycin-induced fibrosis showed sub-pleural and parenchymal foci of inflammation and fibrosis, following treatment with PBS (Figure 10F). In contrast, HYAL treatment reduced bleomycin-induced fibrosis and collagen deposition, as observed in tissues stained for collagen deposition (Figure 10G). Tissue collagen deposition was also measured by picrosirius and a significant decrease was observed after HYAL treatment (Figure 10D). The total soluble collagen assayed by Sircol was also decreased compared with bleomycin treated mice (Figure 10E). These data together showed that HYAL significantly reduced pulmonary fibrosis induced by bleomycin instillation.

## Discussion

These studies of the effects of i.n. HYAL administration revealed two surprising and potentially important new findings. First, we demonstrated that HYAL produced a profound increase in mononuclear cells in the airways and these cells had phenotypic features of MSC-like cells. Second, we showed that treatment with HYAL reduced the bleomycin-induced fibrotic response in the lung parenchyma.

We observed that HYAL induced an unusual profile of cellular migration. Cellular migration into the lung is usually a result of an inflammatory process and involves resident cells, soluble mediators and cells that were recruited to the inflammatory focus [17]. In a typical acute inflammatory setting, neutrophil recruitment is chronologically followed by the arrival of other cells, including macrophages and lymphocytes, depending on the stimulus [18]. However, we did not observe neutrophils within 4 h after HYAL inoculation, which suggests that the enzyme was not a pyogenic inflammatory stimulus *per se*. HYALs comprise a group of enzymes that can be found in a variety of sources [1]. The substrates of HYALs vary according to the source of the enzymes. For example, bovine testes HYAL degrades hyaluronan and chondroitin, while HYALs obtained from *Naja *venom are specific for hyaluronan alone [1]. Considering these differences, we initially hypothesized that HYAL-TS might have distinct actions. However, the *T. serrulatus *HYAL showed the same profile of cellular accumulation produced by bovine testes HYAL. As a result of the similar results, and the difficulty of purifying the enzyme from venom, subsequent experiments were done using bovine testes HYAL.

In this work, inactive HYAL was used to determine whether the mononuclear cell increases were due to alterations in the hyaluronic acid content of the ECM. Either inactive or denatured HYAL showed a markedly reduced capacity to induce mononuclear cell infiltration compared to the active enzyme, establishing that the enzymatic activity was involved in cell migration into the alveolar space. In order to verify the mechanisms involved in this phenomenon, we used diverse pharmacological approaches. First, we used dexamethasone, a potent anti-inflammatory corticosteroid that inhibits leukocyte transmigration, cytokines and lipid mediator release [19]. These experiments showed that dexamethasone reduced the number of cells in the BALF of mice inoculated with HYAL. In addition to the diminished recruitment with inactive HYAL, these data suggest that the cells increased in BALF are not derived from the lung parenchyma but are the result of new cellular migration into the lung *per se*.

Considering the hypothesis that the mononuclear cells were truly recruited following HYAL treatment, we investigated the role of leukotrienes, which are potent leukocyte chemoattractants [20]. Treatment with MK886, an inhibitor of leukotrienes synthesis [21], reduced the number of cells after HYAL inoculation. We also used mice that were unable to produce leukotrienes (5-LO^-/-^) and that also had diminished cellular migration compared to WT animals. In addition, levels of LTB_4 _in lung homogenates of HYAL-exposed mice increased compared with PBS (control) treatment. Taken together, these results suggested that leukotrienes (for example, LTB_4_) are necessary to promote the cellular migration following HYAL treatment. In order to explore the involvement of cyclooxygenase (COX)-derived PGs, we also pre-treated mice with celecoxib, a drug that inhibits COX-2 with five- to 50-fold selectivity over COX-1 [22]. Celecoxib-treated mice had a significantly diminished cellular migration, indicating that PGs could be involved in this process, although we did not find significant increases of PGE_2 _in the lung homogenates obtained from mice inoculated with HYAL. Future studies will be important in the determination of the extent to which other PGs are involved in the effects of HYAL observed in the present study.

An increase in lung cytokines was observed after inoculation with HYAL. However, as mentioned previously, HYAL did not induce neutrophil or eosinophil recruitment. Thus, these paradoxical results suggest that a cell-specific mechanism is involved in MSC recruitment induced by HYAL and that the basis of this selectivity requires further investigation. Here, we demonstrated an increase in IL-10, tumour necrosis factor (TNF)-α, interferon (IFN)-γ, IL-1, IL-2, IL-4, and IL-5 following HYAL. We did not determine the main source of these cytokines, but it is possible that they were produced by diverse lung cell populations including epithelial cells, fibroblasts or endothelial cells [23]. In addition, considering that MSCs have immunosuppressive properties [9] and synthesize paracrine mediators such as cytokines, chemokines and other inflammatory mediators [8,24], it is possible that MSC-like cells might produce these cytokines directly and might also stimulate the immune cells to produce these mediators.

Somewhat unexpectedly, a significant increase in mononuclear cells was observed in the BALF of HYAL-inoculated mice. Markers of a mature cell lineage had diminished expression in these cells. These results indicated that the populations increased in BALF were most likely to be non-differentiated cells. Our hypothesis was that these cells could be MSCs. However, selecting appropriate markers of such cells in mice was challenging. Depending on the source and the strategies used to obtain MSCs, the cellular markers used to describe human MSCs can vary. In fact, there is no consensus about the range of specific markers to be used in order to define a human MSC. To clarify the panel of characteristics necessary for cells to be deemed MSC, *The International Society for Cellular Therapy *recently published minimal criteria for human MSC. This work did not encourage the use of the same criteria for murine MSC because they considered that murine surface markers have not been universally characterized as well as for the human MSC [25]. Meirelles *et al*. considered that the panel of markers may be adjusted in MSC obtained from non- bone-marrow sources [8]. There is no consensus to define a murine MSC and the markers can change depending on the cell stage [26]. In our work, we sought to examine the most common markers used to characterize both human and murine MSC. Bone marrow murine MSC have been characterized as CD11b, CD31, CD34, CD45, CD48, CD90, CD117, CD135 negative and CD29, CD44, CD81, CD105 positive, whereas MSC obtained by immunodepletion express CD9, CD29, CD44, CD81 and CD106 [27].

In our flow cytometry studies, we found that BALF cells from HYAL mice: had positive and high-level expression for some markers (SCA-1, CD44); were positive for others (CD73); and were CD34, CD45 negative, compared to cells derived from PBS-treated animals. In addition, HYAL populations did not present significant differences in the expression of CD3, CD4 and/or CD8, excluding the possibility of these cells being lymphocytes. Although murine MSC do not have highly-specific markers, generally they are CD34^neg^, CD45^pos^, CD73^neg^, CD44^pos ^and SCA-1^pos ^[8,27,28]. Human MSCs have been characterized in the literature with many different markers, depending on the source from which the cells were isolated [29,30]. Indeed some differences related to marker expression might be a consequence of the fact that MSCs are often purified using repeated culture passages. The cells in the present studies were obtained directly from the lung without an additional purification process. Based upon our immunophenotyping data we considered that the cells induced by HYAL instillation were MSC-like cells.

Considering that inhaled HYAL treatment recruited MSC-like cells to the lung, we hypothesized that this enzyme could treat pulmonary disorders currently ameliorated by MSC therapy. Lung disorders such as fibrosis have been considered to be potential targets for MSC therapy, due to the capacity of bone marrow-derived-MSC to differentiate into diverse lung cells [13]. In one study, mice with fibrosis induced by bleomycin had diminished damage and collagen deposition in the lung due to the migration of MSC [16]. In another recent study, MSC from the umbilical cord diminished fibrosis induced by bleomycin [10]. Our results showed that HYAL reduced the histopathological evidence of fibrosis in the lung by decreasing the number of neutrophils, collagen content and the production of TGF-β.

We speculate that MSC-like cells are recruited to the fibrotic lung and differentiate into lung epithelial cells, ameliorating the fibrosis. This idea is supported by the evidence that MSC are able to migrate to injured tissues [13,31]. Bronchiolar stem cells have been defined by the expression of CD45^neg^, CD31^neg^, CD34^neg^, SCA-1^low ^and AF^low ^[32], which are different from the cells induced by HYAL treatment. Additionally, HYAL treatment inhibited TGF-β production. TGF-β is directly involved in the progression of fibrosis and its blockade constitutes an effective treatment of fibrosis. This was demonstrated in a bleomycin model using TGF-β soluble receptor with antifibrotic potential *in vivo *[33]. It is also possible that some of the effects on fibrosis are the result of the direct action of HYAL on HA itself. HA is increased in lung fibrosis and this has been correlated with tissue damage and inflammation [3,5,6]. A high amount of HA in the lungs following pro-fibrotic injury is associated with inflammation due to increases in macrophage locomotion [5]. Another important aspect is related to CD44, the receptor of HA. CD44 has a critical role in resolving lung inflammation, as CD44-deficient mice have persistent inflammation with accumulation of HA fragments and increased activation of TGF-β1 [34]. Thus, we speculate that the decrease in bleomycin-induced lung fibrosis after treatment with HYAL might be due to the actions on HA on the regulation of inflammation, on decreased TGF-β and on the increased recruitment of MSC-like cells to the lung. It should be noted that MSCs have also been reported to differentiate into fibrocytes and contribute to the pathogenesis of pulmonary fibrosis [35] and that bone-marrow derived fibroblasts are thought to be involved in the progression of bleomycin-induced fibrosis [36].

Classical stem cell therapy holds great promise but has some potential negative aspects such as an increased risk for bacterial infections, viral infections and allergic reactions [15,37]. A major obstacle to MSC therapy has been the difficulty of obtaining enough cells to transplant and a lack of standardized and efficient methods to obtain these cells [14]. Regarding MSC use in the treatment of treat lung diseases, these cells represent a promising modality due to their characteristic migration to injured tissue, intrinsic immunosuppressive properties and plasticity to differentiate into mature lung cells required for the lung repair [13].

In summary, we have newly demonstrated that a single dose of HYAL therapy promotes endogenous MSC-like accumulation in the lung and that this intervention might prove beneficial to the therapy for serious diseases such as pulmonary fibrosis.

## Conclusions

These studies demonstrated that the i.n. administration of HYAL produced a profound increase in mononuclear cells, with phenotypic features of MSC-like cells, in the airways. HYAL is a novel and promising tool for the recruitment of autologous MSC-like cells to the lungs in the treatment of pulmonary fibrosis because i.n. HYAL treatment potently blocked bleomycin-induced lung injury and fibrosis while it decreased TGF-β production and collagen deposition. The involvement of lipid mediators in this process might also lead to the identification of new pharmacological tools for ameliorating pulmonary fibrosis.

## Materials and methods

### Animals

C57BL6/6 mice (18-25 g), obtained from the animal facilities of the Faculdade de Ciências Farmacêuticas de Ribeirão Preto, Universidade de São Paulo (FCFRP - USP). Mice lacking 5-LO enzyme gene (5-LO^-/-^) were from The Jackson Laboratory and raised at FCFRP - USP and age-matched male WT (WT-background, strain 129) used as controls. All experiments were approved by the guidelines of the Animal Care Committee of the Universidade de São Paulo (Protocol No. 07.1.782.53.8).

### Reagents

In the majority of the experiments bovine HYAL was used (purchased from Sigma Chemical Co, MO, USA). In some experiments, HYAL was isolated from the venom of *T. serrulatus*, HYAL-TS. MK886 was a gift from Merck Frosst Canada Inc (ON, Canada). Dexamethasone was purchased from Aché^© ^(Laboratórios Farmacêuticos S/A, São Paulo, Brazil). Bleomycin sulphate was purchased from Bristol Myers-Squibb (Blenoxane^®^; São Paulo, Brazil). ELISA antibodies to measure cytokines were from Pharmingen (CA, USA). Neutravidin-horseradish peroxidase was from Perbio Science (Cheshire, UK). K-Blue substrate was from Neogen (KY, USA). Panoptic staining was from Laborclin (Paraná, Brazil). Sircol Collagen Assay was purchased from Biolocor Life Science Assays (Carrickfergus, CA, UK).

### HYAL activity and inactivation

HYAL activity was determined turbidimetrically following protocol described by Pukrittayakamee and modified slightly [38]. For heat inactivation, 100 U of HYAL was heated for 30 min at 60°C [38]. For heat denaturation, HYAL was heated at 95°C for 60 min.

### HYAL experimental design and bleomycin lung injury

Mice were i.n. inoculated with 4, 8 or 16 U of HYAL (Sigma Chemical Co). Controls received PBS. At 4, 24, 48, 96, 240 or 360 h after treatment, animals received carbon dioxide for euthanasia. In some experiments mice received dexamethasone (1 mg/kg weight s.c.); or MK886 (5 mg/kg/0.5 mL by gavage) 1 h before HYAL. Mice that were killed 96 h after stimulus received extra doses every 24 h [39]. Inactive and denaturated HYAL were used in some experiments. Bleomycin sulphate was administered intratracheally 4 U/kg in 0.1 of NaCl 0.9% [16]. After 7 days of bleomycin, animals were treated i.n. with HYAL 16 U or PBS.

### Collection of bronchoalveolar space cells

The trachea was exposed and catheterized. PBS was infused in three 1-mL aliquots in the catheterized trachea and the cells collected. Total cell counts were performed in a Neubauer chamber. Differential counts were obtained using Panoptic staining (similar to Diff-Quick^®^).

### Immunophenotyping of cells by flow cytometry

CD3, CD4, CD8, CD19, CD11c, MAC-3, CD45, CD34, CD73, SCA-1(Ly-6AE) expression were determined through flow cytometry immunostaining protocol using antibody conjugated with fluorochomes (BD Biosciences, NJ, USA). Specific rat IgG2a isotype controls were used to monitor non-specific binding. Stained cells obtained from a BALF pool were washed with PBS containing 2% fetal bovine serum (FBS), pelleted by centrifugation at 400x *g *and fixed with PBS containing 1% (w/v) paraformaldehyde. A total of 10,000 events were acquired (FACSCanto TM; Becton Dickinson, CA, USA), using the FACS Diva for data acquisition and FlowJo for analysis.

### Cytokines, leukotrienes and prostaglandin measurements

LTB_4 _was quantified in lung homogenate using commercial ELISA kits (Cayman Chemical^©^, MI, USA). IL-1, IL-2, IL-4, IL-5, IL-10, IL-12, TNF-α, IFN-γ, TGF-β, MCP-1 and KC were determined by ELISA according to manufacturer instructions (BD Biosciences). Sensitivities were >10 pg/mL.

### Collagen assay

Lung homogenate supernatants were placed in 1.5 mL tubes. Sircol-dye was added, the content of the tubes homogenized for 30 min and centrifuged for 10 min (10,000x *g*). The pellets were dissolved with alkaline reagent. Absorbance was read at 540 nm. The total soluble collagen was determined using a standard curve [40].

### Histopathological studies

Lung were collected and immediately fixed in 10% formalin. Specimens were processed, embedded in paraffin and cut into for to six lm sections and stained with haematoxylin and eosin (H&E). For evaluation of collagen fibres in the pulmonary parenchyma, the slides were stained with Picro Sirius [41]. The surface density of collagen fibres in the lung was determined by optical density by image analysis. The system used consisted of a video camera (Leica Microsystems Ltd, Heebrugg, Switzerland), applied to a Leica microscope DMR (Leica, Microsystems GmbH, Wetzlar, Germany) attached to a computer. The images were processed by software Leica QWin software (Leica Microsystems Image Solutions, Cambridge, UK). The thresholds for collagen fibres were established for each slide, after adjusting the contrast to a point at which the fibres were easily identified as red bands. Bronchovascular bundles were carefully avoided during the measurements. Ten randomly selected inflammatory foci presented in the pulmonary parenchyma were considered for each group at 400x. The total area examined for group was 0.8 mm^2^. The means was calculated and the values expressed as a percentage.

### Statistical analysis

Data are expressed as mean ± standard error of mean. Statistical variations were determined by analysis of variance (ANOVA) and Student's *t*-test. Values of *P *< 0.05 were considered significant.

## Abbreviations

BALF: bronchoalveolar fluid; ECM: extracellular matrix; FBS: fetal bovine serum; HA: hyaluronic acid; HYAL: hyaluronidase; IL: interleukin; i.n.: intranasal inoculation; LPS: lipopolysaccharide; MSC: mesenchymal stem cell; PBS: phosphate buffered saline; PG: prostaglandin; SCA: stem cell antigen; TGF: transforming growth factor.

## Conflict of interest statement

We certify that there is no conflict of interest with any financial organization regarding the material discussed in the manuscript.

## Authors' contributions

CSB conducted the majority of experiments and wrote the manuscript; PATP contributed to bleomycin assays; SGR conducted the histological analyses; ECA provided HYAL-TS and edited the manuscript; SVC edited the manuscript; DMA assisted with writing and editing the manuscript; LHF conceived of, designed, corrected the manuscript and supervised the project. All authors discussed the results and implications and commented on the manuscript at all stages, read and approved the final document.

## Supplementary Material

Additional File 1 **Figure S1. The expression of CD3, CD4, CD8, CD19, MAC-3 and CD11c in bronchoalveolar fluid (BALF) cells**. (A) Gate 1 is a representative picture showing forward/side scatters dot-plot of BALF cells obtained from phosphate buffered saline (PBS) treatment (black contour-plot) overlaid upon hyaluronidase (HYAL) exposure (grey dot-plot), as well as the gate population highlighted in red. Histogram overlays were done using the gated population highlighted in red, where the open histogram is the isotype; the grey filled histogram represents PBS treatment; red filled histogram represents HYAL. Figure depicts a representative analysis from five independent experiments. The mean (B) and median (C) expression of markers is altered by HYAL. PBS values were considered 100%. (D) Gate 2 is a representative picture showing forward/side scatters dot-plot of BALF cells obtained from PBS treatment (black contour-plot) overlaid upon HYAL exposure (grey dot-plot), as well as the gate of interest highlighted in red. Histogram overlays were done using the gated population highlighted in red, where the open histogram is for the isotype control; grey filled histogram represents PBS treatment; red filled histogram represents HYAL. The figure depicts a representative analysis from five independent experiments. The mean (E) and median (F) expression of markers is altered by HYAL. PBS values were considered 100%. Click here for file

Additional File 2 **Figure S2. Cells induced by 16 U of bovine testicular hyaluronidase (HYAL) to the bronchoaveolar fluid (BALF) of C57Bl/6 mice**. (A) Gate 3 is a representative picture showing forward/side scatters dot-plot of BALF cells obtained from phosphate buffered saline (PBS} treatment (black contour-plot) overlaid upon HYAL exposure (grey dot-plot), as well as the gate of interest highlighted in red. Histograms overlays were done using the gated population highlighted in red; open histogram = isotype control; grey filled histogram = PBS treatment; red filled histogram = HYAL treatment. The figure depicts a representative analysis from five independent experiments. (B) Gate 3 is a representative picture showing forward/side scatters dot-plot of BALF cells obtained from PBS treatment (black) overlaid upon the HYAL exposure (red). The figure depicts a representative analysis from five independent experiments. The mean (C) and median (D) expression of markers is altered by HYAL. PBS values were considered 100%. (E) Gate 4 is a representative picture showing forward/side scatters dot-plot of BALF cells obtained from PBS treatment (black contour-plot) overlaid upon HYAL exposure (grey dot-plot), as well as the gate of interest highlighted in red. Histograms overlays were done using the gated population highlighted in red; open histogram = isotype control; grey filled histogram = PBS treatment; red filled histogram = HYAL treatment. The figure depicts a representative analysis from five independent experiments. (F) Gate 4 is a representative picture showing forward/side scatters dot-plot of BALF cells obtained from PBS treatment (black) overlaid upon HYAL exposure (red). The figure depicts a representative analysis from five independent experiments. The mean (G) and median (H) expression of markers was altered by HYAL, presented as the percentage of variation compared to control. The controls mice were considered 100%. Click here for file

Additional File 3 **Figure S3. Effects of hyaluronidase (HYAL) on bleomycin-induced inflammation in the lung**. (A) Neutrophils numbers in the bleomycin inoculated mice were significantly increased 7 days after inoculation (*P *> 0.001). Values represent means ± standard error of mean (SEM); *n *= 5, Student *t *test was used (B) mononuclear cells in bleomycin inoculated mice were significantly increased 7 days after inoculation (*P *> 0.0001). Values represent means ± SEM; *n *= 5, test *t *was used. (C) MCP-1 concentration in supernatant of lung tissue homogenate was determined by ELISA. Values represent means ± SEM; *n *= 5, *P *= 0.9526 compared with bleomycin; Student *t *test was used. (D) KC concentration in supernatant of lung tissue homogenate was determined by ELISA. Values represent means ± SEM; *n *= 5, *P *= 0.3063 compared with bleomycin; Student *t *test was used. Click here for file
