# Supplementary figures and images for: Hyaluronidase recruits mesenchymal-like cells to the lung and ameliorates fibrosis
Source: Fibrogenesis Tissue Repair. 2011 Jan 13;4:3. doi: 10.1186/1755-1536-4-3 (PMC3035036; doi:10.1186/1755-1536-4-3)

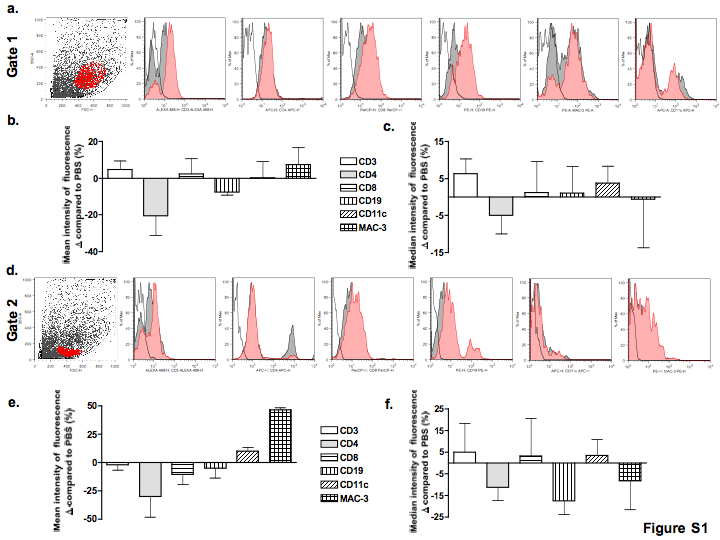

Supplement: Additional File 1 — Figure S1. The expression of CD3, CD4, CD8, CD19, MAC-3 and CD11c in bronchoalveolar fluid (BALF) cells. (A) Gate 1 is a representative picture showing forward/side scatters dot-plot of BALF cells obtained from phosphate buffered saline (PBS) treatment (black contour-plot) overlaid upon hyaluronidase (HYAL) exposure (grey dot-plot), as well as the gate population highlighted in red. Histogram overlays were done using the gated population highlighted in red, where the open histogram is the isotype; the grey filled histogram represents PBS treatment; red filled histogram represents HYAL. Figure depicts a representative analysis from five independent experiments. The mean (B) and median (C) expression of markers is altered by HYAL. PBS values were considered 100%. (D) Gate 2 is a representative picture showing forward/side scatters dot-plot of BALF cells obtained from PBS treatment (black contour-plot) overlaid upon HYAL exposure (grey dot-plot), as well as the gate of interest highlighted in red. Histogram overlays were done using the gated population highlighted in red, where the open histogram is for the isotype control; grey filled histogram represents PBS treatment; red filled histogram represents HYAL. The figure depicts a representative analysis from five independent experiments. The mean (E) and median (F) expression of markers is altered by HYAL. PBS values were considered 100%. [file 1755-1536-4-3-S1.TIFF]

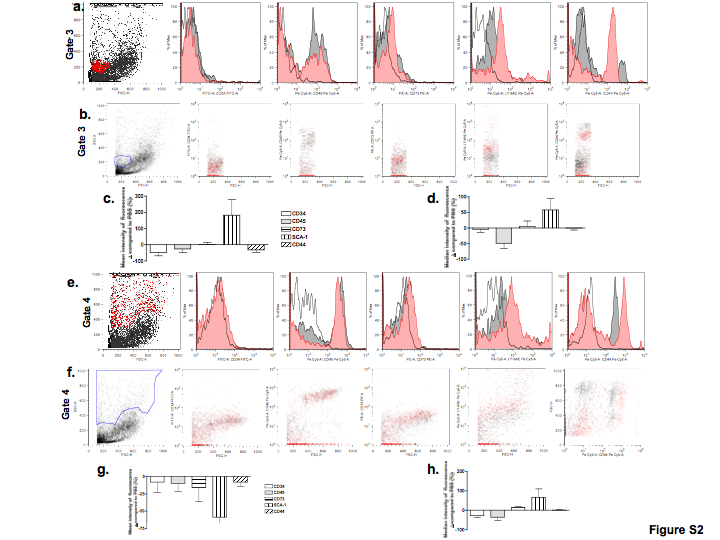

Supplement: Additional File 2 — Figure S2. Cells induced by 16 U of bovine testicular hyaluronidase (HYAL) to the bronchoaveolar fluid (BALF) of C57Bl/6 mice. (A) Gate 3 is a representative picture showing forward/side scatters dot-plot of BALF cells obtained from phosphate buffered saline (PBS} treatment (black contour-plot) overlaid upon HYAL exposure (grey dot-plot), as well as the gate of interest highlighted in red. Histograms overlays were done using the gated population highlighted in red; open histogram = isotype control; grey filled histogram = PBS treatment; red filled histogram = HYAL treatment. The figure depicts a representative analysis from five independent experiments. (B) Gate 3 is a representative picture showing forward/side scatters dot-plot of BALF cells obtained from PBS treatment (black) overlaid upon the HYAL exposure (red). The figure depicts a representative analysis from five independent experiments. The mean (C) and median (D) expression of markers is altered by HYAL. PBS values were considered 100%. (E) Gate 4 is a representative picture showing forward/side scatters dot-plot of BALF cells obtained from PBS treatment (black contour-plot) overlaid upon HYAL exposure (grey dot-plot), as well as the gate of interest highlighted in red. Histograms overlays were done using the gated population highlighted in red; open histogram = isotype control; grey filled histogram = PBS treatment; red filled histogram = HYAL treatment. The figure depicts a representative analysis from five independent experiments. (F) Gate 4 is a representative picture showing forward/side scatters dot-plot of BALF cells obtained from PBS treatment (black) overlaid upon HYAL exposure (red). The figure depicts a representative analysis from five independent experiments. The mean (G) and median (H) expression of markers was altered by HYAL, presented as the percentage of variation compared to control. The controls mice were considered 100%. [file 1755-1536-4-3-S2.TIFF]

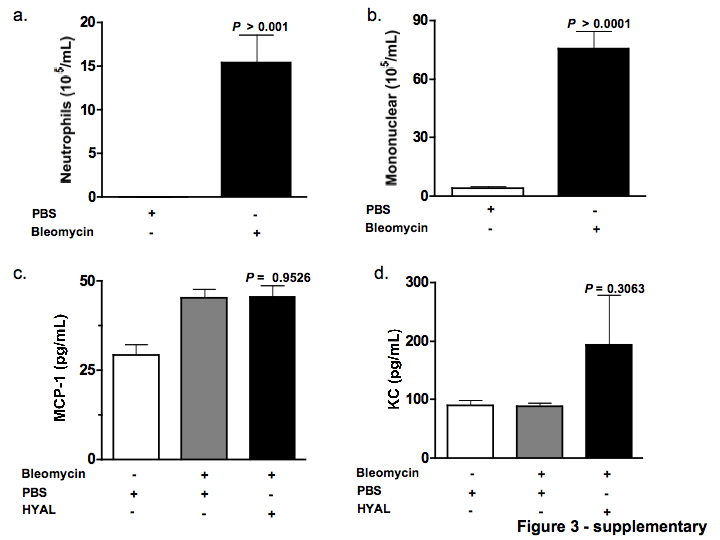

Supplement: Additional File 3 — Figure S3. Effects of hyaluronidase (HYAL) on bleomycin-induced inflammation in the lung. (A) Neutrophils numbers in the bleomycin inoculated mice were significantly increased 7 days after inoculation (P > 0.001). Values represent means ± standard error of mean (SEM); n = 5, Student t test was used (B) mononuclear cells in bleomycin inoculated mice were significantly increased 7 days after inoculation (P > 0.0001). Values represent means ± SEM; n = 5, test t was used. (C) MCP-1 concentration in supernatant of lung tissue homogenate was determined by ELISA. Values represent means ± SEM; n = 5, P = 0.9526 compared with bleomycin; Student t test was used. (D) KC concentration in supernatant of lung tissue homogenate was determined by ELISA. Values represent means ± SEM; n = 5, P = 0.3063 compared with bleomycin; Student t test was used. [file 1755-1536-4-3-S3.TIFF]
